# Arousal in Nocturnal Consciousness: How Dream- and Sleep-Experiences May Inform Us of Poor Sleep Quality, Stress, and Psychopathology

**DOI:** 10.3389/fpsyg.2017.00733

**Published:** 2017-05-10

**Authors:** Nirit Soffer-Dudek

**Affiliations:** Consciousness and Psychopathology Laboratory, Department of Psychology, Ben-Gurion University of the NegevBeer-Sheva, Israel

**Keywords:** sleep experiences, dreaming, sleep quality, psychopathology, distress, nightmares, hypnagogic hallucinations, sleep-wake disorders

## Abstract

The term “sleep experiences,” coined by Watson (2001), denotes an array of unusual nocturnal consciousness phenomena; for example, nightmares, vivid or recurrent dreams, hypnagogic hallucinations, dreams of falling or flying, confusional arousals, and lucid dreams. Excluding the latter, these experiences reflect a single factor of atypical oneiric cognitions (“general sleep experiences”). The current study is an opinionated mini-review on the associations of this factor—measured with the Iowa sleep experiences survey (ISES, Watson, 2001)—with psychopathological symptoms and stress. Findings support a strong relation between psychological distress and general sleep experiences. It is suggested that that they should be viewed as a sleep disturbance; they seem to represent involuntary intrusions of wakefulness into sleep, resulting in aroused sleep. These intrusions may stem from excessively thin boundaries between consciousness states (e.g., “transliminality”), or, conversely, they may follow an attempt at disconnecting mental elements (e.g., dissociation), which paradoxically results in a “rebound effect.” The extent to which unusual dreaming is experienced as intrusive, rather than controlled, may explain why general sleep experiences are related to psychopathology, whereas lucid dreams are related to psychological resilience. In conclusion, the exploration of the interplay between psychopathology and sleep should be expanded from focusing almost exclusively on quantitative aspects (e.g., sleep efficiency, latency) to including qualitative conscious experiences which may reflect poor sleep quality. Taking into account nocturnal consciousness—including unusual dreaming and permeable sleep-wake boundaries—may unveil rich information on night-time emotional states and broaden our definition of poor sleep quality.

A vast body of literature shows that sleep problems are related to an array of psychopathological disorders, including depression, bipolar disorder, anxiety disorders, posttraumatic stress disorder (PTSD), obsessive-compulsive disorder, schizophrenia, dissociation, alcoholism, eating disorders, attention deficit hyperactivity disorder, dementia, and autism (Benca et al., 1992; Chouinard et al., 2004; Spoormaker and Montgomery, 2008; Cortese et al., 2009; Sedky et al., 2014; van Heugten–van der Kloet et al., 2014a; Díaz-Román et al., 2015; Nota et al., 2015). Most studies on psychopathology and sleep assess sleep quality or disruption in terms of one's ability to fall asleep with ease and sleep through the night uninterrupted. Therefore, research has mostly relied on quantitative measures. These include, for example, total/true sleep time, sleep efficiency, sleep latency, wake after sleep onset, and number of awakenings (e.g., Elrod and Hood, 2015; Ng et al., 2015). Indeed, these variables play a central role in psychopathology and have the advantage of possible objective assessment (e.g., polysomnography, actigraphy).

However, the focus on quantitative measures is intertwined with a certain neglect of subjective—or qualitative—aspects of the sleeper's consciousness, such as dream characteristics. This relative neglect is evident in the Diagnostic and Statistical Manual of Mental Disorders, Fifth Edition (DSM-5; American Psychiatric Association, 2013); out of numerous disorders of sleep-wake processes, spanning problems with the amount and timing of sleep, breathing-disordered sleep, and parasomnias (e.g., sleepwalking), only one, namely, nightmare disorder (ND), is primarily concerned with an alteration in dreaming. Similarly, while symptoms involving quantitative sleep alterations appear in numerous diagnoses in other chapters of the manual (e.g., mood disorders), qualitative nocturnal experiences are hardly addressed. Other than “recurrent distressing dreams” related to traumatic content in the diagnosis of PTSD (American Psychiatric Association, 2013), dreaming does not play a major role in the DSM-5. Thus, although the field of Psychology is interested not just in behavior but also in subjective experience, it seems that current views of mental health do not tend to take into account characteristics of *nocturnal consciousness* when defining or diagnosing psychopathology. Granted, dream research suffers from the problem of subjectivity, and dream reports may be unreliable and open to bias (see Schwartz and Maquet, 2002, for a review of methodological problems in dream research). Yet, this problem may be reduced by conducting rigorous studies exploring relationships between dream- and sleep-experiences and emotion with daily diaries (e.g., Soffer-Dudek and Shahar, 2011), or by lexical analysis of dreams (Schwartz and Maquet, 2002). I assert that paying attention to nocturnal subjective experience is informative and worthwhile. The current investigation is a qualitative mini-review on the relation between psychopathology and a trait representing unique nocturnal consciousness.

## Nocturnal consciousness and its importance for the exploration of psychopathology

By using the term “nocturnal consciousness,” rather than “dreaming,” I refer to subjective experience throughout various sleep stages and sleep-wake transitions. Specifically, I wish to emphasize: (1) that I am not merely considering the content of dreams, but also their structural or formal aspects, such as repetitiveness, vividness, and bizarreness; and (2) that the review is not limited to typical REM dreams, but also includes non-REM mentation, which may be experienced as “thoughts” (McNamara et al., 2010), as well as sleep-wake transition phenomena such as sleep paralysis, hypnagogic hallucinations, and confusion upon awakening. Notably, the present review is meant to supplement and extend the already-established literature on non-REM sleep-wake disorders (sleepwalking, night terrors). These disorders, representing arousal in sleep, are strongly related to stress and psychopathology (Schenck and Mahowald, 2005).

Do alterations in nocturnal consciousness play a significant role in psychopathology? Indeed, there is ample evidence that dream characteristics are associated with several psychopathological symptoms. For example, nightmares are related to emotional distress, trauma, and personality disorders (e.g., Zadra and Donderi, 2000; Levin and Nielsen, 2009; Davis et al., 2011; Casement and Swanson, 2012; Schredl, 2016), and share a specific association with suicidality (Cukrowicz et al., 2006; Sjöström et al., 2007; Pigeon et al., 2012). Suicidality is also related to a reduction in dream-like quality of dream content reports between the first and second half of the night (Agargun and Cartwright, 2003). Alterations in dreaming seem to play a part in psychosis as well (D'Agostino et al., 2012, 2013; Cavallotti et al., 2014). In fact, the dream sleep stage (i.e., REM sleep), has been suggested as a model for schizophrenia (Gottesmann, 2006), and there is some evidence that psychosis may be conceptualized as sleeping mentation entering the waking state (e.g., Sponheim et al., 1994). The various nocturnal consciousness characteristics that have been related to stress, negative emotion, or psychopathological symptoms include—but are not limited to—traumatic and non-traumatic nightmares (e.g., van der Kolk et al., 1984; Hartmann, 1998; Levin, 1998; Cukrowicz et al., 2006; Pigeon et al., 2012), recurrent dreams (Zadra et al., 1997–1998; Duke and Davidson, 2002), falling dreams (e.g., Kroth et al., 2002; Schredl, 2007), dream bizarreness (e.g., Cavallotti et al., 2014), and hypnagogic hallucinations (Ohayon et al., 1996; Ohayon, 2000; Koffel, 2011).

Additionally, in recent years a broad trait of unique dream characteristics has been strongly and repeatedly linked with distress. In 2001, David Watson analyzed items addressing various aspects of unique dream and sleep-wake transition experiences, resulting in two factors for the “Iowa Sleep Experiences Survey” (ISES; Watson, 2001). Fifteen items loaded onto a non-specific factor, which includes an array of unusual dreams and sleep-wake phenomena (henceforth labeled general sleep experiences; GSEs; Watson, 2001), such as remembering dreams, nightmares, recurrent dreams, vivid dreams, dreams of flying or falling, confusion between dreams and reality, false awakenings (i.e., awakening within the dream), dreams of dying, and hypnagogic hallucinations. Three items loaded on a separate factor, pertaining to the experience of lucidity in dreams (i.e., awareness of the fact of dreaming, while maintaining the sleep state, and control over dream events). This subscale (LDs) is moderately related to GSEs (e.g., *r* = 0.42, *p* < 0.001, Soffer-Dudek and Shahar, 2009), suggesting that they are not identical. GSEs (but not LDs) have repeatedly been associated with negative emotionality, stress, and psychopathological symptoms. Notably, studies that explored relationships of psychopathology with individual ISES items (e.g., Watson, 2001; Soffer-Dudek et al., 2011a) found relations with most items, suggesting that the relation of GSEs with psychopathology does not stem specifically from the nightmare item or any other single item. Table 1 reviews research that examines the relationship between GSEs (as measures with the ISES) along with measure(s) of negative emotion, stress, or psychopathological symptoms^1^.

**Table 1:** **Studies demonstrating relationships between general sleep experiences (GSEs) as assessed by Watson's (2001) Iowa sleep experiences survey (ISES), and psychopathology-related constructs such as negative affectivity, stress, and psychological symptoms**.

**Study**	**Sample characteristics**	**Effect size and statistical significance for each related construct**	**Relation type**
Watson, 2001	Sample 1: *N* = 471 undergraduate students, ~59% females.	Neuroticism: *r* = 0.28^**^; Dissociation: *r* = 0.42^**^–0.57^**^; Schizotypy: *r* = 0.36^**^–0.47^**^.	Cross-sectional correlations.
	Sample 2: *N* = 457 undergraduate students, ~64% females.	Neuroticism: *r* = 0.24^**^; Dissociation: *r* = 0.44^**^–0.54^**^; Schizotypy: *r* = 0.31^**^–0.45^**^.	
Watson, 2003	*N* = 169 undergraduate students, 73.37% females.	Neuroticism/Negative Emotionality: *r* = 0.18^*^; Dissociation: *r* = 0.30^**^–0.52^**^; Schizotypy: *r* = 0.20^**^–0.36^**^ (note: this study used an earlier version of the ISES, which included items pertaining to lucid dreaming in the same factor score. Thus, correlations with psychopathology are lower, compared to studies using the GSEs factor).	Cross-sectional correlations (note: the neuroticism measure was administered 2 months before the other measures).
Giesbrecht and Merckelbach, 2004	*N* = 94 undergraduate students, 68.09% females. *M*_age_ = 21.25, *SD*_age_ = 2.16, age range: 18–27.	Dissociation: *r* = 0.38^**^.	Cross-sectional correlation.
Fassler et al., 2006	*N* = 163 undergraduate students, 66.87% females. *M*_age_ = 19.77, *SD*_age_ = 1.96, age range: 18–35.	Negative affect: *r* = 0.37^**^; Dissociation: *r* = 0.35^**^.	Cross-sectional correlations.
Giesbrecht and Merckelbach, 2006a	*N* = 205 undergraduate students, 68.29% females. *M*_age_ = 19.4, *SD*_age_ = 1.75, age range: 17–26.	Dissociation: *r* = 0.35^**^.	Cross-sectional correlation.
Giesbrecht and Merckelbach, 2006b	*N* = 87 undergraduate students.	Dissociation: *r* = 0.37^**^.	Cross-sectional correlation.
Giesbrecht et al., 2006	*N* = 67 undergraduate students, 88.06% females. *M*_age_ = 21.1, *SD*_age_ = 2.68, age range: 18–31.	Dissociation: *r* = 0.47^**^–0.55^**^.	Cross-sectional correlation.
Koffel and Watson, 2009	Sample 1: *N* = 376 undergraduate students (note: Sample 2 is not reported because no correlations were reported for that sample).	Negative affectivity: *r* = 0.27; obsessive-compulsive symptoms: *r* = 0.34; Dissociation: *r* = 0.45 (Note: statistical significance levels were not presented in this study; however, the authors stated that the correlation coefficient of GSEs with dissociation was significantly stronger than the other two correlation coefficients).	Cross-sectional correlations.
Soffer-Dudek and Shahar, 2009	*N* = 203 undergraduate students, 85% females. *M*_age_ = 23.6, *SD*_age_ = 1.86, age range: 17–33.	Dissociation: *r* = 0.33^**^–0.44^**^; Psychological distress symptoms: *r* = 0.34^**^–0.40^**^; Stressful life events: *r* = 0.23^**^−0.30^**^.	Cross-sectional correlations.
		An elevation over the course of 3 months in stressful life events was related to a parallel elevation in GSEs (β = 0.34^**^).	Correlations extended in time (predicting change with change over a 3 month interval).
		Psychological distress at Time 1 predicted an elevation in GSEs (β = 0.14^**^) while dissociation (β = 0.05) and stressful life events (β = −0.08) did not.	Prospective-longitudinal prediction of GSEs over the course of 3 months.
Soffer-Dudek and Shahar, 2010	*N* = 91 participants recently exposed to missile terror attacks, through direct exposure or media and relational exposure. They were taken from the pool of participants from Soffer-Dudek and Shahar (2009) which was conducted 3 years prior to this study. Approx. 80% females. *M*_age_ = 23.8, age range: 21–33.	Psychological distress symptoms: *r* = 0.50^**^; Exposure to terror through the media: *r* = 0.26^*^; Physical exposure: *r* = −0.21^*^ (reverse effect); physical location: *r* = −0.33^**^ (reverse effect); Stress-related exposure: *r* = 0.19; Relational exposure: *r* = −0.04.	Cross-sectional correlations.
		Exposure to the attacks through the media (as reported at Time 2) predicted an elevation in GSEs (β = 0.27^**^). Physical location had a reverse effect (β = −0.26^**^), suggesting that the closer to the area of attacks one resided, the more GSEs decreased. (Note: sleep loss due to nocturnal alarms was not controlled for in this study, which may have confounded the association). Time 1 psychological distress (β = 0.16), relational exposure (β = 0.16), physical exposure (β = −0.16), and stress-related exposure (β = −0.04) did not have a statistically significant effect on change in GSEs.	Prediction of longitudinal change in GSEs over the course of 3 years.
Kucukgoncu et al., 2010	*N* = 200 undergraduate students with no current psychiatric complaints. 52.76% females. *M*_age_ = 23.07, *SD*_age_ = 2.12, range = 10.	Dissociation: *r* = 0.49^**^−0.56^**^	Cross-sectional correlation.
Soffer-Dudek and Shahar, 2011	*N* = 200 undergraduate students, 77.50% females. *M*_age_ = 23.36, *SD*_age_ = 1.40, age range: 18–28.	Dissociation: *r* = 0.29^**^–0.53^**^; Psychological distress symptoms: *r* = 0.50^**^; depressive symptoms: *r* = 0.44^**^; Stressful life events: *r* = 0.44^**^−0.46^**^.	Cross-sectional correlations.
	*N* = 60, a random subset from the participant pool described above. 71.67% females. *M*_age_ = 23.54, *SD*_age_ = 1.16, age range: 21–26.	Daily stress ratings: *semi-partial R^2^* = 0.08^*^; Trait dissociation: *semi-partial R^2^* = 0.12^**^; state dissociation (*semi-partial R^2^* = 0.00), psychological distress (*semi-partial R^2^* = 0.00) and life stress (*semi-partial R^2^* = 0.00) did not have a statistically significant effect (Note: standardized effect sizes for multilevel modeling analyses were not originally published. They were calculated here based on the degrees of freedom and the *t*-value which were included in the published data. For each relevant predictor effect, a standardized effect size of explained variance was calculated, namely semi-partial *R*^2^; Edwards et al., 2008).	Longitudinal prediction of change in daily GSEs across 14 days.
Soffer-Dudek et al., 2011a	*N* = 19 outpatients of a mental health clinic, 13 of them diagnosed with schizophrenia or related diagnoses, 4 with bipolar disorder, and 2 with anxiety disorders (52.63% females. *M*_age_ = 37.55, *SD*_age_ = 13.53), and *N* = 26 controls from the community (53.85% females. *M*_age_ = 38.58, *SD*_age_ = 13.74).	Psychological distress symptoms: *r* = 0.34–0.35; Stress: *r* = 0.00–0.46^*^; Daytime dysfunction: *r* = 0.36–0.61^**^; Illness intrusiveness: *r* = 0.49^*^.	Cross-sectional correlations within each group separately.
		GSEs of outpatients: *M* = 3.17, *SD* = 1.23; GSEs of controls:*M* = 2.30, *SD* = 0.84; comparison: *t*_(43)_ = 2.82^**^.	Comparison of means between groups.
van der Kloet et al., 2012a (Note: this study did not use the ISES *per se* but used subscales of a sleep measure pertaining to nightmares and narcoleptic symptoms to mimic the ISES and explicitly aimed to test Watson's predictions)	*N* = 195 mixed (mainly depressed) inpatients undergoing eclectic psychotherapy for 6–8 weeks. Approx. 43% females. Approx. *M*_age_ = 44.2, *SD*_age_ = 11.5, age range: 18–74.	*For the narcolepsy subscale:* Dissociation: *r* = 0.29^**^; Psychopathology composite: *r* = 0.23^**^.	Correlations extended in time: correlations between change variables (across 6–8 weeks).
		*For the nightmares subscale:* Dissociation: *r* = 0.01; Psychopathology composite: *r* = −0.06.	
Van Der Kloet et al., 2013 (Note: this study relied on the general ISES score rather than the subscale score for GSEs)	*N* = 45 inpatients diagnosed with primary insomnia, 62.22% females, *M*_age_ = 41.5, *SD*_age_ = 13.68, age range: 17–78.	Psychological distress symptoms: *r* = 0.37^*^; Dissociation: *r* = 0.40^**^.	Cross-sectional correlations.
Knox and Lynn, 2014	*N* = 86 undergraduate students (63.95% females) in the out-of-context condition, and *N* = 87 undergraduate students (58.62% females) in the in-context condition. *Median*_age_ = 18, age range: 17–24.	*Out of context condition:* Negative emotion: *r* = 0.29^**^; Schizotypy: *r* = 0.25^*^–0.38^**^; Dissociation: *r* = 0.36^**^.	Cross-sectional correlations in each condition separately
		*In-context condition:* Negative emotion: *r* = −0.10–0.08; Schizotypy: *r* = −0.09–0.42^**^; Dissociation: *r* = 0.42^**^.	
van Heugten–van der Kloet et al., 2014a	Patients (100% female) with dissociative identity disorder (DID; *n* = 12, *M*_age_ = 42, *SD*_age_ = 11.8) and post-traumatic stress disorder (PTSD; *n* = 27, *M*_age_ = 42, *SD*_age_ = 13.1), and healthy female controls (*n* = 55, *M*_age_ = 42, *SD*_age_ = 13.1).	Dissociation: *r* = 0.63^**^	Cross-sectional correlation across groups.
		GSEs of DID: *M* = 62.50, *SD* = 17.71; GSEs of PTSD: *M* = 57.93, *SD* = 19.96; GSEs of controls: *M* = 34.11, *SD* = 12.48; comparison: *F* = 30.10^**^ (the authors state that DID significantly differed from controls and PTSD significantly differed from controls, with no significant difference between DID and PTSD).	Comparison of means between groups.
Van Heugten–van der Kloet et al., 2014b	*N* = 139 undergraduate students, 87.77% females. *M*_age_ = 21.4, age range: 17–32.	Dissociation: *r* = 0.39^*^(after transformation of the dissociation score: *r* = 0.41^**^).	Cross-sectional correlation.
van Heugten–van der Kloet et al., 2015a	*N* = 72 participants older than 18 who chose to enter a photo contest, ~77% females. *M*_age_ = 35.8, *SD*_age_ = 16.9.	Dissociation: *r* = 0.40^*^–0.54^*^.	Cross-sectional correlations.
van Heugten–van der Kloet et al., 2015b (Note: this study relied on the general ISES score rather than the subscale score for GSEs).	*N* = 56 undergraduate students with no serious mental disease or sleep problems. 76.79% females. *M*_age_ = 20.7, *SD*_age_ = 2.33, age range: 18–29.	Dissociation: *r* = 0.45^**^–0.55^**^.	Cross-sectional correlations.
		Dissociation: −0.21–0.27; Mood: 0.34 (Note: statistical significance in this study is reported only at the *p* < 0.01 level; the deterioration in mood following sleep loss seems to be significant at the *p* < 0.05 level but it cannot be ascertained based on the published data).	Correlation of ISES score with the change score of psychopathology following a period of sleep loss.
Merckelbach et al., 2015	*N* = 22 inpatients hospitalized at a psychiatric facility specializing in trauma (participants were diagnosed with PTSD, dissociative disorders), mood disorders, and borderline personality disorder. 81.82% females, *M*_age_ = 38.8, *SD*_age_ = 10.15, age range: 20–59.	Dissociation: 0.40–0.59^**^.	Cross-sectional correlations.
Watson et al., 2015 *(Note: this study used a composite score encompassing both the ISES and additional scales assessing unusual sleep experiences)*	*N* = 406 adults, 68.49% females. *M*_age_ = 44.9, *SD*_age_ = 13.3, age range: 18–74. Nearly half of the sample (*N* = 188) received treatment for mental health issues.	Panic: *r*_SR_ = 0.67, *r*_ID_ = 0.33; Posttraumatic stress: *r*_SR_ = 0.65, *r*_ID_ = 0.40; Generalized anxiety: *r*_SR_ = 0.61, *r*_ID_ = 0.44; Depression: *r*_SR_ = 0.58, *r*_ID_ = 0.39–0.48; Obsessive-compulsive: *r*_SR_ = 0.53, *r*_ID_ = 0.35; Social anxiety: *r*_SR_ = 0.47, *r*_ID_ = 0.30; Agoraphobia: *r*_SR_ = 0.45, *r*_ID_ = 0.38; Substance use: *r*_SR_ = 0.12–0.24, *r*_ID_ = 0.10–0.18; Bipolar: *r*_SR_ = 0.20–0.50, *r*_ID_ = 0.46; Schizotypy/psychosis: *r*_SR_ = 0.24–0.65, *r*_ID_ = 0.42–0.51; Dissociation: *r*_SR_ = 0.69 (no *r*_ID_ for dissociation). (Note: Statistical significance of the correlations is not reported in this study. SR, self-report. ID, interview diagnosis).	Cross-sectional correlations with self-report scales and (polyserial correlations) with interview diagnoses.
Soffer-Dudek, 2016	*N* = 53 participants recently exposed to missile terror attacks, through direct exposure or media and relational exposure. They were taken from the pool of participants from Soffer-Dudek and Shahar (2011) which was conducted 3 years prior to this study. 79.25% females. *M*_age_ = 26.23, age range: 24–29.	Psychological distress symptoms: *r* = 0.47^**^; Dissociation: *r* = 0.42^**^; Exposure to terror through the media: *r* = 0.33^*^; Physical exposure: *r* = –0.14; Stress-related exposure: *r* = 0.15; Relational exposure: *r* = 0.04.	Cross-sectional correlations.
		Time 1 GSEs predicted an elevation in psychological distress symptoms: β = 0.34^**^, even when controlling for the effect of the degree of exposure to terrorism. This effect was replicated when reanalyzing data from Soffer-Dudek and Shahar (2010): β = 0.23^*^.	GSEs predicting, prospectively-longitudinally, change in psychological symptoms over the course of 3 years.
Vissia et al., 2016	Patients (100% female) with genuine dissociative identity disorder (DID-G; *n* = 17) and post-traumatic stress disorder (PTSD; *n* = 16), and healthy female controls (*n* = 16), as well as DID simulators (DID-S; *n* = 16). All participants’ ages ranged from 18 to 65 and were matched between groups.	GSEs of DID-G: *M* = 57.88, *SD* = 16.83; GSEs of PTSD: *M* = 50.81, *SD* = 14.81; GSEs of controls: *M* = 28.63, *SD* = 10.22; GSEs of DID-S: *M* = 38.07, *SD* = 11.91; comparison: *H*_(3)_ = 27.80^**^ (DID-G significantly differed from controls and PTSD significantly differed from controls, with no significant difference between DID-G and PTSD. DID-G significantly differed from DID-S).	Comparison of means between groups.

Age and gender are reported only when such data are available.

* p < 0.05, two-tailed.

** *p ≤ 0.01 or lower, two-tailed*.

As evident from the table, GSEs are positively associated with various psychopathological symptoms. The first types of symptoms associated with GSEs were dissociative experiences (Watson, 2001, 2003; Giesbrecht and Merckelbach, 2004, 2006a; Fassler et al., 2006) and schizotypy (Watson, 2001, 2003). On the basis of these relations, Watson concluded that the constructs form a common domain involving unusual cognitions and perceptions in waking and in sleep (Watson, 2001). Koffel and Watson (2009) further clarified that GSEs are characteristic of a psychopathological tendency for oddity, including magical ideation, suspiciousness, ideas of reference, unusual perceptions, and odd speech, behaviors, and beliefs. They reviewed findings of higher correlations of GSEs with dissociation (Koffel and Watson, 2009) and with dissociation and schizotypy (Watson, 2001), compared with negative affectivity and obsessive-compulsive symptoms (Koffel and Watson, 2009) or with neuroticism (Watson, 2001; see also Knox and Lynn, 2014). Hence, they suggested that GSEs are specific to dissociation and schizotypy, whereas insomnia and lassitude are specific to depression and anxiety (Koffel and Watson, 2009; Koffel, 2011).

However, Fassler et al. (2006) found that effect sizes of GSEs with dissociation were similar in magnitude to those with negative emotion. Subsequent research on GSEs pointed to strong non-specific associations with a wide array of psychological distress symptoms (Soffer-Dudek and Shahar, 2009, 2010, 2011; Watson et al., 2015; Soffer-Dudek, 2016). Moreover, this relationship has been extended from student samples to clinical samples (Soffer-Dudek et al., 2011a; Watson et al., 2015), including not only self-report methods but also using rigorous interview-based diagnoses (Watson et al., 2015). GSEs do not merely relate concurrently to distress, but also longitudinally. Specifically, psychological symptoms (a general distress score, as well as several subscales: somatization, obsessive-compulsive symptoms, depression, hostility, paranoid ideation, and psychoticism) prospectively predicted an elevation in GSEs across a 3 month interval (Soffer-Dudek and Shahar, 2009). Moreover, baseline GSEs were a potent prospective predictor of an elevation in psychopathological symptoms following exposure to terror 3 years later (Soffer-Dudek, 2016). Studies using other measures focusing on specific unusual sleep events also support these relations with distress. For example, in a study on adults reporting childhood trauma, McNally and Clancy (2005) found that sleep paralysis is related to both depression and dissociation, with similar effect sizes. In addition, nightmares show moderate to strong correlations with depression and anxiety in student and psychiatric samples (e.g., Koffel and Watson, 2010).

The two views of GSEs, namely, as an altered consciousness trait pertaining to unusual cognitions and as a manifestation of psychological distress, are not necessarily contradictory. One study integrated them by demonstrating a symmetrical diathesis-stress interaction, according to which the effect of trait dissociation on daily GSEs was present only in the face of high daily stress, and the effect of daily stress on GSEs existed only among those high in trait dissociation (Soffer-Dudek and Shahar, 2011). Thus, it seems that for individuals who are prone to experience unusual cognitions, psychological distress may be manifested in alterations in nocturnal consciousness.

## GSEs as disrupted sleep

The relationship of GSEs with various forms of psychopathological distress, reviewed in Table 1, suggests that GSEs may represent a form of distressed sleep. This raises a question, namely: Should there be some correlation between GSEs and traditional (quantitative) measures of disturbed sleep quality? Or are GSEs an entirely different, independent form of sleep disturbance? Research has been equivocal on this matter. Several studies did find a relation (Kucukgoncu et al., 2010; Soffer-Dudek and Shahar, 2011; van Heugten–van der Kloet et al., 2014a, 2015a,b; Soffer-Dudek, 2016). However, others did not (Watson, 2003; Van Der Kloet et al., 2013). Knox and Lynn (2014) found it only in one of their two samples. These null findings may be rooted in the fact that Van Der Kloet et al. (2013) and Watson (2003) used the total ISES score, which included LDs, and that Watson (2003) and Knox and Lynn (2014) did not separate sleep quality from sleep duration. In a rigorous daily study, Soffer-Dudek and Shahar (2011) showed that elevated GSEs were related to poor sleep quality, but also to long sleep duration (see also van Heugten–van der Kloet et al., 2015a, for somewhat similar results).

GSEs as sleep disruptions have been conceptualized as arousal and hypervigilance permeating the dream state (Soffer-Dudek and Shahar, 2011). This conceptualization is similar to the classic view of disrupted sleep, according to which, vigilance to threat pulls one away from sleep and toward waking (e.g., Dahl, 1996); except that it does not view sleep and waking as *mutually exclusive* states, but rather posits that arousal may exert its influence on consciousness even without waking up the sleeper. Undeniably, non-REM parasomnias (e.g., sleepwalking) are hybrid sleep-wake states; GSEs may represent the corresponding REM sleep phenomena (e.g., elevated dream recall, nightmares, hypnagogic hallucinations, intensely kinesthetic dreams, vivid dreams, confusional arousals, false awakenings, and recurrent dreams, characterized by increased access to memory). Indeed, individuals suffering from insomnia show greater brain metabolism during sleep (Nofzinger et al., 2004), and higher frequency EEG activity during sleep onset and non-REM sleep (e.g., Perlis et al., 2001), especially in the face of stress (Hall et al., 2007). Moreover, stress and worry carry prolonged cardiovascular effects into sleep, independent of sleep quality, which have been labeled “unconscious worry” (Brosschot et al., 2007). Importantly, some individuals seem to be especially capable of lingering in mixed sleep-wake states, including dissociative experiences and parasomnias (Mahowald and Schenck, 2001; Giesbrecht et al., 2008; Koffel and Watson, 2009; van der Kloet et al., 2012b). Dissociation and GSEs may reflect a reciprocal process in which sleep and waking intrude into one another. The conceptualization of GSEs as an intrusion of waking into sleep is concordant with the finding that among patients with severe psychopathology, GSEs were related to their experience of the mental illness as intrusive (Soffer-Dudek et al., 2011a).

## The paradox of intrusion: notes on the traits that lead to GSEs

Enduring the intrusion of waking within dreaming suggests increased fusion and association between consciousness states. Watson (2001) proposed that the trait responsible for the continuum of unusual cognitions may be “thin boundaries”—a tendency for ideas, emotions, memories, and sensations to associate or merge (Hartmann, 1991), or “transliminality”—a similar tendency for psychological material to pass fluidly between consciousness thresholds (Lange et al., 2000). Indeed, GSEs are related to transliminalty (Soffer-Dudek and Shahar, 2009) and synaesthesia (Terhune, 2009). Yet, dissociation, which is closely related to GSEs, is a state of *separation* of mental elements, as can be inferred from its name. Dissociative absorption, for example, is defined as the total allocation of attention to a stimulus while being oblivious to surrounding stimuli (Soffer-Dudek et al., 2015). How can these definitions harmoniously co-exist?

A possible explanation for this seeming discrepancy is the dream rebound effect of avoidance. Specifically, actively attempting not to think about a stimulus (e.g., a white bear), results in increased dreaming about that object (Wegner et al., 2004; Taylor and Bryant, 2007; Schmidt and Gendolla, 2008; Kröner-Borowik et al., 2013; Malinowski, 2016), and especially when facing cognitive load (Bryant et al., 2011). Ironically, suppressing thoughts leads to attempts at monitoring those thoughts, which in turn generate intrusions, resulting in an increase in the occurrence of the thoughts (Wegner, 1994; Wenzlaff and Wegner, 2000). This pertains to the core dynamics of PTSD: The more the individual exerts effort in avoiding thoughts of the trauma, the more they will appear uninvited, as dissociative flashbacks and nightmares. These processes may similarly govern the occurrence of GSEs: Intrusion of arousal into dreaming may be rooted not only in a tendency for increased *association* of mental elements (i.e., thin boundaries, enhanced continuity between waking and dreaming), but also in a tendency for increased *avoidance* and attempts at separating consciousness elements from each other (e.g., memories and emotions), such as dissociative mechanisms, which ironically also result in increased intrusion.

The idea that GSEs are experienced as an uncontrolled intrusion into sleep may be the explanation for the relative lack of findings on LDs and psychopathology. Specifically, LDs are also a “mixed state” between waking and sleeping (Mahowald and Schenck, 2001; Voss et al., 2009); they are even related to blurred boundaries between reality and fantasy (Corlett et al., 2014). However, they do not show the same strong and persistent relation with psychopathology and stress that GSEs do (e.g., Soffer-Dudek and Shahar, 2009; Soffer-Dudek, 2016). This difference may stem from the subjective feeling of control characterizing many LDs, in which the plot of the dream may be volitionally influenced by the dreamer. Indeed, LDs have been related to an internal locus of control (Blagrove and Tucker, 1994; Blagrove and Hartnell, 2000; Patrick and Durndell, 2004) and to psychological resilience in the face of exposure to terror (Soffer-Dudek et al., 2011b).

In contrast, GSEs seem to resemble uncontrolled nocturnal rumination: the uninhibited lingering of distress in the sleeper's consciousness. Indeed, during stressful military conflicts, GSEs are related to a somewhat ruminative inclination to keep watching the news, even though it causes the watcher distress (Soffer-Dudek and Shahar, 2010; Soffer-Dudek, 2016). This tendency to hold on to distress, whether during daytime or nighttime, may be the explanation for some enigmatic findings. Specifically, Soffer-Dudek and Sadeh (2013) found that self-reported unusual dreaming in children predicted an increase in parental ratings of the children's behavior problems (internalizing and externalizing) from age 10 to 12; this suggested that unusual dreaming identified somewhat covert distress or *potential* for psychopathology, undetectable by parents at age 10. Similarly, Soffer-Dudek (2016) also demonstrated that GSEs may reflect covert aspects of distress. Specifically, baseline GSEs prospectively predicted an elevation in psychological distress following exposure to terror attacks 3 years later, over and above the participants' own account of how much they were exposed and distressed by the attacks. GSEs may identify potential future distress, because they represent the inclination to ruminatively and uncontrollably linger in a mixed sleep-wake state, an inclination which places the individual at risk for psychopathology^2^.

## Limitations, future directions, and conclusions

A major methodological limitation of the research reviewed is that it is largely questionnaire-based; hence relationships may suffer from inflation due to biased reporting and shared method variance. Notably, however, daily diaries (e.g., Soffer-Dudek and Shahar, 2011), are less biased than self-reporting on the past month or year because reporting is proximal to the experience, thus less distorted by memory. Future research should also examine daily emotion using ecological momentary assessment and relations with GSEs, to investigate directional relationships, perhaps in conjunction with polysomnography. In addition, scoring dream content using blind raters may provide a less biased approach to the assessment of the relation between dream themes and psychopathology (e.g., Schredl and Engelhardt, 2001). Still, the field would benefit from the inclusion of basic and experimental research. For example, although psychiatric medications affect dreaming, such examinations are scarce (Tribl et al., 2013). Also, although interventions of lucidity with nightmare re-scripting are probably useful in reducing distress (Gavie and Revonsuo, 2010), such research is in its infancy.

Additionally, more work is needed in order to integrate the literatures on nocturnal consciousness and non-REM parasomnias; these also represent mixed sleep-wake states, relate to dissociation (Mahowald and Schenck, 2001), and to psychopathological distress (Schenck and Mahowald, 2005). It is yet to be determined why stress should affect deep sleep in one individual (bringing about unremembered behavioral episodes), and dreaming consciousness in another (resulting in unusual nocturnal cognitions). Possibly, the extent of dissociative tendencies may be responsible; this remains an issue for future research to explore. Future studies should also work to integrate quantitative and qualitative nocturnal characteristics. For example, severely depressed individuals have more REM at the beginning of the night, but less of it during morning-time (Vogel et al., 1980), when dreaming is most frequent; Indeed, their dreaming is impoverished (Cartwright, 2010), suggesting that a complete quantitative-qualitative picture may elucidate psychopathology.

Finally, another limitation of some of the studies reviewed is their correlational design, limiting the ability to draw causal conclusions. However, several studies employed prospective-longitudinal paradigms, assessing change and examining directionality; these studies showed that GSEs both *follow* and prospectively *predict* change in psychopathology. Also, one study implemented time-lag analysis on daily data, showing that GSEs followed stress, rather than vice versa (Soffer-Dudek and Shahar, 2011). Further, research should establish whether GSEs are a consequence of emotional distress, a cause, or both. Several researchers have assigned an active role of emotion regulation and modification to dreaming (e.g., Hartmann, 1998; Levin and Nielsen, 2009; Cartwright, 2010) and more generally, to REM sleep (e.g., Genzel et al., 2015). GSEs may either reflect impaired regulation, or act as a dysregulating force in and of themselves. For example, dreams may engender predictive coding (Kirov, 2016), functioning as a “virtual-reality generator” (Hobson et al., 2014); GSEs may actively impede this mechanism, by introducing external arousal and awareness instead of fully delusional and endogenous REM dreams. Indeed, emotional arousal has been conceptualized as energetic impingement, which brings about both psychopathological symptoms and dream imagery, as the brain's attempt to reduce free energy (Hopkins, 2016); GSEs may reflect impinged-upon dreaming, which joins forces with waking mechanisms to create psychopathological disorders.

In conclusion, some unusual nocturnal experiences seem to be “waking” processes entering sleep states. They increase in frequency during stressful periods, and may be viewed as sleep disruptions. Some of these phenomena are widely accepted as sleep disturbances (e.g., nightmares), whereas others are regarded as esoteric or are hardly regarded at all (e.g., falling dreams, false awakenings). Although GSEs and other dream characteristics are associated with psychopathological symptoms, qualitative aspects of sleep are not included in most diagnostic criteria and rarely routinely assessed. Unusual nocturnal consciousness characteristics are an important way to explore sleep impairment in psychopathology. This relatively under-researched field may lead to novel insights regarding nocturnal emotional states and the underpinnings of consciousness, and may contribute to understanding the specificity of sleep impairment in different psychopathological disorders.

## Author contributions

NSD is solely responsible for the conception, literature review, and writing of this paper.

### Conflict of interest statement

The author declares that the research was conducted in the absence of any commercial or financial relationships that could be construed as a potential conflict of interest.

## Footnotes

^1^See van der Kloet et al. (2012b) for a review on the ISES and dissociation.

^2^The notion that individuals are unaware of the connection between their GSEs and distress is concordant with the finding that the relationship between sleep experiences and dissociation does not stem from symptom exaggeration (Merckelbach et al., 2015).

## Abbreviations

GSEs: general sleep experiences
LDs: lucid dreams
PTSD: posttraumatic stress disorder
ND: nightmare disorder.
